# Effects and Molecular Mechanisms of Heat-Killed Postbiotic *Enterococcus faecalis* EF-2001 on Muscle Volume and Grip Strength in Dexamethasone-Induced Muscle Atrophy in SD Rats

**DOI:** 10.3390/ijms27021105

**Published:** 2026-01-22

**Authors:** Jin-Ho Lee, Kwon-Il Han, Eunwoo Jeong, Juyeong Moon, Min-ah Kim, Bon Seo Koo, Yura Lee, Sunhwa Baek, Han Sung Kim, Tack-Joong Kim

**Affiliations:** 1 Division of Biological Science and Technology, Yonsei University, Wonju 26493, Republic of Korea; drlogos@naver.com (J.-H.L.); jew0108@naver.com (E.J.); mjy@yonsei.ac.kr (J.M.); mina1218@yonsei.ac.kr (M.-a.K.); kbs1349@yonsei.ac.kr (B.S.K.); 2 Research & Development Center, bereum Co., Ltd., Wonju 26362, Republic of Korea; kihan@bereum.com (K.-I.H.); lyr@bereum.com (Y.L.); an20112246@bereum.com (S.B.); 3 Department of Biomedical Engineering, Yonsei University, Wonju 26493, Republic of Korea; hanskim@yonsei.ac.kr

**Keywords:** *Enterococcus faecalis*, EF-2001, postbiotic, muscle atrophy, C2C12, grip strength

## Abstract

The interaction between the gut microbiota and human health has gained increasing recognition, accelerating advances in microbiome research. While early studies have emphasized probiotics, concerns regarding antibiotic resistance and adverse effects, such as sepsis, have shifted research interest towards heat-treated microbial cells or postbiotics. This study investigated the therapeutic potential of heat-killed postbiotic *Enterococcus faecalis* EF-2001—one of the most widely used postbiotics worldwide—for the prevention and treatment of muscle atrophy. In vitro, mouse C2C12 myotubes were pretreated with heat-killed postbiotic EF-2001 (50–500 μg/mL) for 48 h and then treated with dexamethasone (100 μM) to induce muscle atrophy. In vivo, male Sprague Dawley rats were treated with low-dose (3 mg/kg) and high-dose (30 mg/kg) EF-2001 for efficacy studies. Heat-killed postbiotic EF-2001 attenuated cellular and DNA damage in dexamethasone-induced C2C12 myotubes. Specifically, heat-killed postbiotic EF-2001 increased AKT phosphorylation while suppressing Atrogin-1 expression, thereby alleviating muscle atrophy. In a Sprague Dawley rat model, heat-killed postbiotic EF-2001 significantly reduced dexamethasone-induced muscle loss by regulating muscle atrophy-associated signaling pathways, including Atrogin-1 expression. Collectively, these findings demonstrate that heat-killed EF-2001 alleviates dexamethasone-induced muscle atrophy and support its potential as a postbiotic. This study provides a solid foundation for future human clinical studies by establishing preclinical evidence for the biological activity of heat-killed EF-2001.

## 1. Introduction

According to international consensus (EWGSOP2) definitions, sarcopenia is defined as a progressive and generalized skeletal muscle disorder involving the loss of muscle mass, muscle strength, and physical performance. These alterations may arise from multiple factors, including inadequate nutrition, aging, genetic predisposition, and physical inactivity [1,2]. Physiological atrophy occurs as a result of muscle disuse, whereas neurogenic atrophy arises from nerve damage or neurological disorders [3]. In the context of aging, muscle atrophy is commonly observed and is a key pathological feature of sarcopenia, an age related skeletal muscle disorder characterized by concomitant declines in muscle strength and physical performance [4].

Sarcopenia is a progressive skeletal muscle disorder characterized by loss of muscle mass, strength, and physical performance with advancing age. It is associated with serious health outcomes, including falls, fractures, and diminished physical independence, ultimately leading to a substantial decline in quality of life [5,6]. In 2016, the International Classification of Diseases, Tenth Revision, Clinical Modification (ICD-10-CM) recognized sarcopenia as a disease (ICD-10-CM M62.84), and in 2021, the Korean Standard Classification of Diseases, Eighth Revision (KCD-8) also added sarcopenia as a clinical condition (diagnostic code M62.5) [7,8]. With a global increase in the aging population, sarcopenia has emerged as an important medical and social concern [9,10]. However, to date, no pharmacological therapy has been approved for its treatment. Non-pharmacological interventions, such as resistance exercise and nutritional management, remain the primary strategies for its prevention and management [11,12].

The development of sarcopenia is closely associated with age-related biological alterations in the skeletal muscle. The activation of the ubiquitin–proteasome system (UPS) plays a central role in regulating muscle protein degradation. In this pathway, the E3 ubiquitin ligases muscle RING finger-1 (MuRF1) and atrogin-1 (MAFbx) target structural and contractile proteins for ubiquitination, thereby promoting proteolysis and accelerating muscle atrophy [13,14,15,16]. The expression of these atrogenes is further influenced by glucocorticoid signaling through the glucocorticoid receptor (GR), which enhances catabolic processes in muscle tissue.

In addition to increased proteolysis, aged skeletal muscles exhibit decreased protein synthesis, mitochondrial dysfunction, oxidative stress, and chronic low-grade inflammation—all of which synergistically contribute to the progression of muscle wasting. The experimental model of dexamethasone (DEX)-induced muscle atrophy is well-established and widely utilized in vitro and in vivo. DEX is a corticosteroid used to treat rheumatic conditions, skin diseases, allergies, and asthma [5,17]. Furthermore, DEX treatment increased the expression of proteasome-related enzymes while suppressing the expression of myogenic differentiation factors, thereby accelerating muscle atrophy [18,19]. Owing to these characteristics, the DEX-induced muscle atrophy model enables the investigation of molecular and functional features associated with sarcopenia within a relatively short period and provides high experimental reproducibility compared with aging models.

DEX is a synthetic glucocorticoid that activates GR-mediated pathways, leading to the upregulation of MuRF1 and atrogin-1 expression [18,19]. This model effectively mimics muscle atrophy and provides a reproducible tool for studying the molecular mechanisms related to sarcopenia-associated catabolic pathways [20,21,22,23]. Activation of AKT signaling has been recognized as a key strategy for suppressing muscle protein degradation through inhibition of FOXO transcription factors, whereas mTOR primarily promotes protein synthesis. Activation of this pathway stimulates muscle protein synthesis and myogenic differentiation while simultaneously inhibiting the expression of atrogin-1, thereby alleviating muscle atrophy [11].

Growing evidence suggests that the gut–muscle axis and microbiota-derived molecules contribute to the regulation of skeletal muscle metabolism and musculoskeletal regeneration. Probiotics and their derived extracellular vesicles (EVs) have recently emerged as potential therapeutic candidates for mitigating muscle atrophy and enhancing muscle function [24,25]. These microbiota-based strategies highlight the expanding role of microbial interventions in sarcopenia research. Compared with live probiotic supplementation, postbiotics offer several advantages, including enhanced safety, greater stability during storage and processing, and reduced risk of microbial translocation. Such properties improve their translational feasibility for applications targeting muscle atrophy and age-related muscle decline. Recently, increasing attention has been directed toward postbiotics as an emerging alternative to probiotics for the prevention and management of muscle loss [26,27]. Postbiotics are considered safer and more stable than live probiotics and exhibit a variety of bioactive properties, including antioxidant, anti-inflammatory, and immunomodulatory effects [28,29].

In this study, we investigated the potential protective effects of EF-2001, a postbiotic preparation composed of heat-killed postbiotics and their metabolites, against DEX-induced muscle atrophy.

## 2. Results

### 2.1. Effect of Heat-Killed Postbiotic EF-2001 on the Attenuation of DNA Damage and Cell Viability in Dexamethasone-Induced C2C12 Myotubes

**Figure 1:**
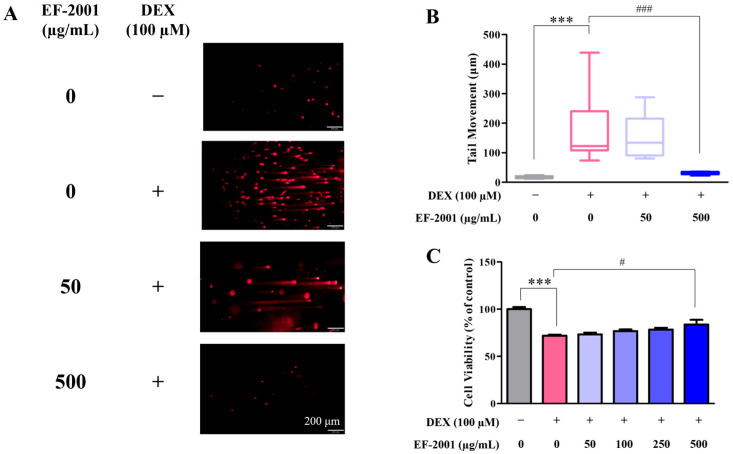
Effects of heat-killed postbiotic EF-2001 on DEX-induced DNA damage and cell viability in C2C12 myotubes. (**A**) Cells were pretreated with heat-killed postbiotic EF-2001 for 48 h on day 5 and treated, with or without 0.5 mM DEX, for 24 h on day 7. scale bar: 200 μm. (**B**) DNA damage score. DNA damage is observed in the comet’s tail, whereas intact DNA remains in the comet’s head. (**C**) C2C12 myotubes was pretreated with various concentrations of heat-killed postbiotic EF-2001 (50, 100, 250, and 500 μg/mL) for 48 h, after which cell viability was measured after treatment with DEX for 24 h by MTT assay. Data are represented as means ± SEM (*n* = 3). *** *p* < 0.001 vs. control group. ^#^ *p* < 0.05, ^###^ *p* < 0.001 vs. DEX-induced control group.

Single-strand breaks in DNA were increased by DEX treatment compared to those in the control. DEX-induced DNA damage resulted in the formation of long tails during gel electrophoresis, whereas the amount of damaged DNA decreased with increasing concentrations of heat-killed postbiotic EF-2001 (50 or 500 ug/mL) (Figure 1A,B). The Comet assay revealed that tail moment in the control group (without heat-killed postbiotic EF-2001 or DEX treatment) was 16.9 ± 23.81. In contrast, DEX treatment markedly increased the tail moment to 179.01 ± 67.32, indicating enhanced DNA damage. Heat-killed postbiotic EF-2001 treatment reduced the tail moment in a concentration-dependent manner, showing 155.51 ± 49.36 at 50 µg/mL and 31.26 ± 14.71 at 500 µg/mL. In addition, cell viability assay showed that heat-killed postbiotic EF-2001 significantly alleviated DEX-induced C2C12 myotube damage (Figure 1C). The decreased cell viability observed in the DEX-treated group was dose-dependently restored by heat-killed postbiotic EF-2001, and at 500 μg/mL, the viability was comparable to that of the control group. The cell viability in the control group (without heat-killed postbiotic EF-2001 or DEX treatment) was 100 ± 5.9%. In contrast, the DEX-induced group showed a significantly decreased cell viability of 71.99 ± 2.87%, corresponding to a 28% reduction compared to the control group, indicating that DEX treatment markedly affected cell viability. Subsequently, treatment with heat-killed postbiotic EF-2001 restored cell viability in a concentration-dependent manner, showing 73.37 ± 4.85% at 50 µg/mL, 76.79 ± 4.82% at 100 µg/mL, 78.46 ± 4.88% at 250 µg/mL, and 83.85 ± 13.21% at 500 µg/mL.

### 2.2. Effect of Heat-Killed Postbiotic EF-2001 in Preventing Muscle Atrophy in Dexamethasone-Induced C2C12 Myotubes

**Figure 2:**
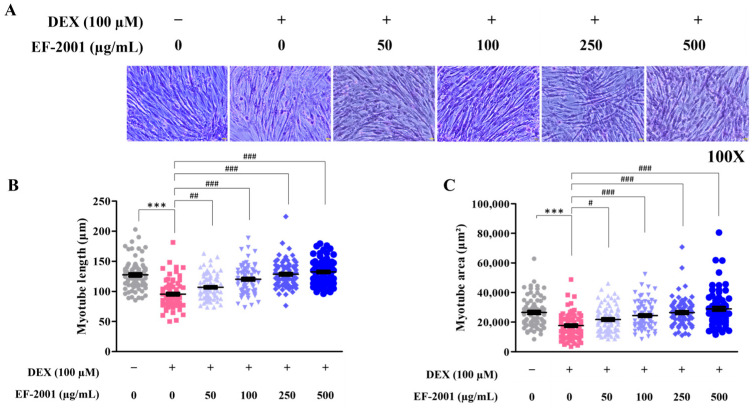
Effects of heat-killed postbiotic EF-2001 against DEX-induced cell damage in C2C12 myotubes and morphological analysis. (**A**) Representative images of Giemsa stained C2C12 myotubes (**B**) Myotube length of cells was calculated on day 7. Myotube length from randomly selected fields were quantified using ImageJ (version 1.8.0.170). Data are represented as means ± SEM (*n* = 80). (**C**) Myotube area of cells was measured on day 7. Myotube diameters from randomly selected fields were quantified using ImageJ, accounting for magnification of the microscope. Data are represented as means ± SEM (*n* = 80). *** *p* < 0.001 vs. control group. ^#^ *p* < 0.05, ^##^ *p* < 0.01, ^###^ *p* < 0.001 vs. DEX-induced group.

To evaluate the effects of heat-killed postbiotic EF-2001 on myotube differentiation, Giemsa staining was performed on C2C12 cells, and the myotube length and area were recorded. Heat-killed postbiotic EF-2001 was administered at various concentrations for 48 h on days five and six of myotube differentiation (Figure 2A). DEX was administered on day seven to induce muscle atrophy. Giemsa staining showed that myotube length and area increased in the group treated with heat-killed postbiotic EF-2001, even after dexamethasone-induced muscle cell damage, confirming a dose-dependent protective effect (Figure 2B,C). In the length measurement, treatment with DEX caused a 25.1% reduction compared with the control group. In contrast, heat-killed postbiotic EF-2001 administration increased the length by 9.0%, 19.5%, 25.9%, and 29.0% at 50, 100, 250, and 500 µg/mL, respectively. In the area measurement, treatment with DEX caused a 33.3% reduction compared with the control group. In contrast, treatment of heat-killed postbiotic EF-2001 increased the area by 15.6%, 25.7%, 33.1%, and 42.6% at 50, 100, 250, and 500 μg/mL, respectively.

### 2.3. Effect of Heat-Killed Postbiotic EF-2001 on AKT Signaling Pathway in Dexamethasone-Induced C2C12 Myotubes

**Figure 3:**
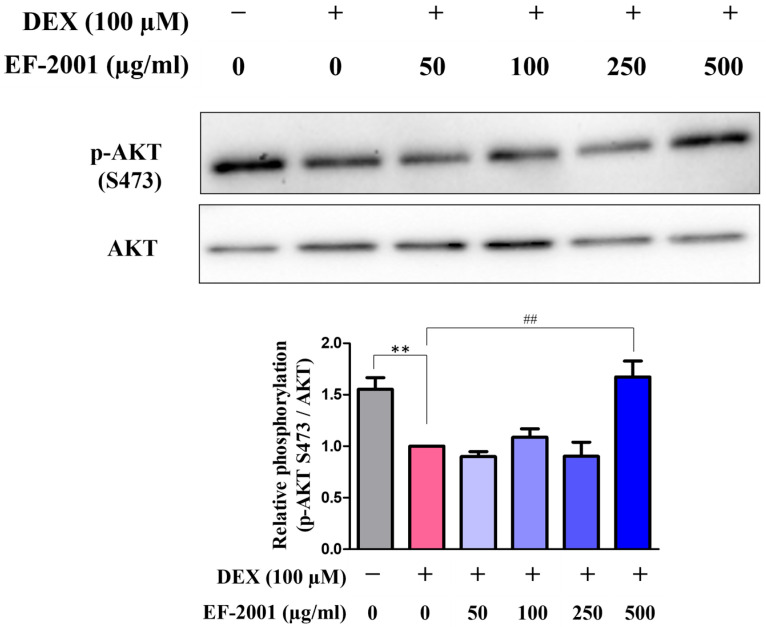
Effects of heat-killed postbiotic EF-2001 on phosphorylation of AKT in DEX-induced C2C12 myotubes. Representative Western blot result of p-AKT (S473). Blots were quantified by densitometry and are expressed relative to the control group. Data are presented as means ± SEM (*n* = 4). ** *p* < 0.01, and vs. control group. ^##^ *p* < 0.01 vs. DEX-induced control group.

To investigate whether heat-killed postbiotic EF-2001 affects the phosphorylation of AKT in differentiated muscle cells, myotubes were treated with various concentrations of heat-killed postbiotic EF-2001 (50, 100, 250, 500 μg/mL) for 48 h, followed by muscle damage induction with DEX. DEX treatment significantly decreased p-AKT levels. However, pretreatment with heat-killed postbiotic EF-2001 significantly attenuated this decrease (Figure 3). Quantitative analysis showed that phosphorylation levels were reduced to 55.2% in the DEX-treated group compared to the control, whereas heat-killed postbiotic EF-2001 (500 μg/mL) treatment restored phosphorylation to 67.2%.

### 2.4. Effect of Heat-Killed Postbiotic EF-2001 on the Downregulation of Muscle Atrophy-Related Protein in Dexamethasone-Induced C2C12 Myotubes

**Figure 4:**
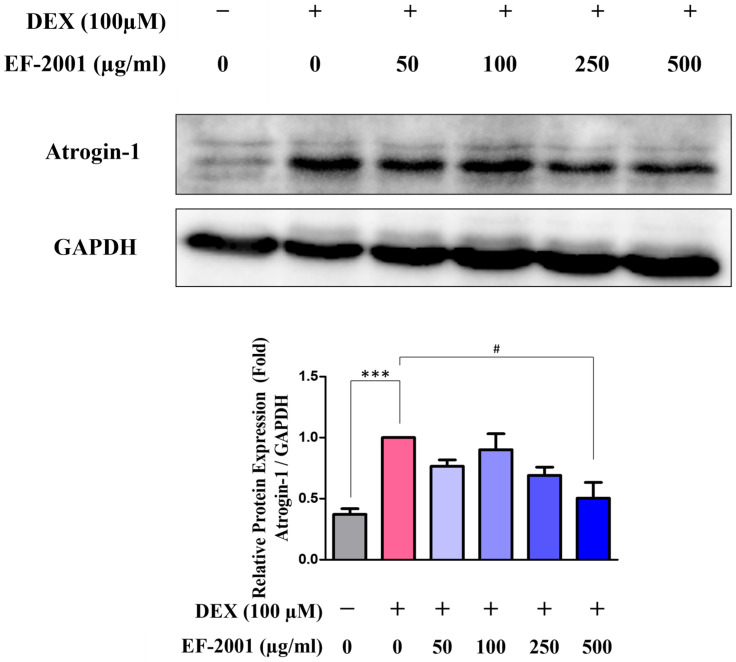
Effects of heat-killed postbiotic EF-2001 on expression of muscle atrophy related protein in DEX-induced C2C12 myotubes. Representative western Blots were quantified by densitometry and are expressed relative to the control group. The protein expression of atrogin-1 on DEX-induced C2C12 myotubes. Protein expression levels were normalized to GAPDH for quantification. Data are presented as means ± SEM (*n* = 4). *** *p* < 0.001, and vs. control group. ^#^ *p* < 0.05 vs. DEX-induced control group.

To further examine the effect of heat-killed postbiotic EF-2001 on DEX-induced biomarkers associated with muscle protein degradation were analyzed. In particular, protein expression levels of atrogin-1, a well-established marker of muscle atrophy, were assessed in DEX-treated myotubes. However, in groups pretreated with heat-killed postbiotic EF-2001, atrogin-1 expression decreased in a dose-dependent manner with heat-killed postbiotic EF-2001 to 500 μg/mL (Figure 4). The expression level of atrogin-1 was significantly elevated by 62.8% in the DEX-treated group compared in the control group. However, treatment with heat-killed postbiotic EF-2001 at 500 µg/mL significantly suppressed atrogin-1 expression by 49.6% relative to the DEX group.

### 2.5. Effect of Heat-Killed Postbiotic EF-2001 on Body Weight and Grip Strength in Dexamethasone-Induced Muscle Atrophy in Rat Model

**Figure 5:**
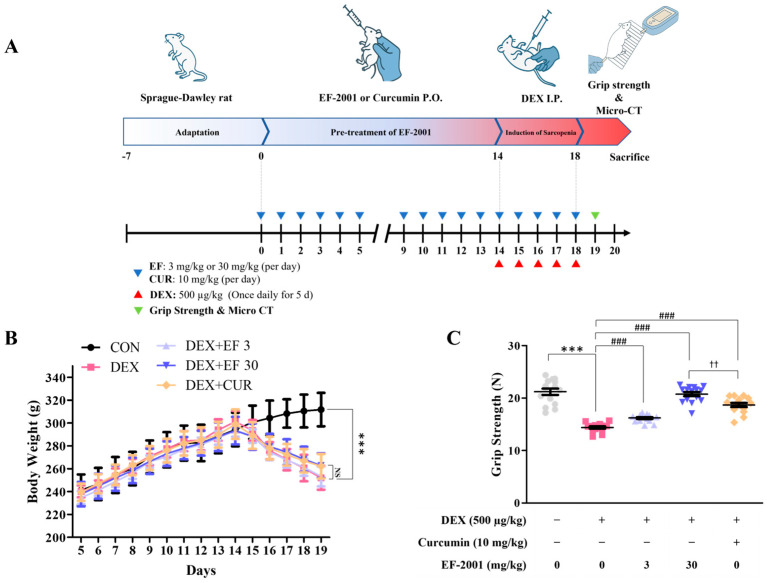
Effects of heat-killed postbiotic EF-2001 on body weight and grip strength in DEX-induced muscle atrophy SD rats. (**A**) Schematic for heat-killed postbiotic EF-2001 administration and experimentation on SD rats. Micro-CT scans were performed three times for each rat, and the final scan was conducted before sacrifice. (**B**) Body weight changes in experimental animals after heat-killed postbiotic EF-2001 administration. The heat-killed postbiotic EF-2001 or curcumin were orally administered per day. Data are represented as mean ± SEM (*n* = 15 per group). *** *p* < 0.001 vs. DEX-induced control group. (**C**) Forelimb grip strength in the study groups before sacrifice. Each dot represents the average of more than five tests per animal. Data are presented as means ± SEM (*n* = 15 per group). *** *p* < 0.001, and vs. control group. ^###^ *p* < 0.001, and vs. DEX-induced control group. ^††^ *p* < 0.01, EF 30 vs. Curcumin group; NS, not significant.

To establish a DEX-induced muscle atrophy model, rats were divided into a control group (untreated group) or a DEX-induced group. Rats were orally administered refined water or heat-killed postbiotic EF-2001 in water at each dose per day, as scheduled. Four DEX-induced subgroups (orally administered distilled water, 3 mg/kg heat-killed postbiotic EF-2001 in water, 30 mg/kg heat-killed postbiotic EF-2001 in water, and 10 mg/kg curcumin in water) were used to examine the effects of EF-2001 or curcumin administration on DEX-induced muscle atrophy (Figure 5A). Curcumin, a polyphenolic compound derived from Curcuma longa, ameliorates dexamethasone-induced muscle atrophy by reducing oxidative stress, suppressing the expression of atrophy-related genes such as MuRF1 and Atrogin-1 signaling pathways [30,31,32,33,34,35]. Therefore, in the present study, curcumin was selected as a positive control to benchmark the efficacy of postbiotics in the prevention of muscle atrophy. Rats injected with DEX showed a significant reduction in body weight compared with those injected with saline. However, the average body weight loss was similar in DEX-injected rats receiving 3 mg/kg, 30 mg/kg EF-2001, or 10 mg/kg curcumin compared to those receiving water only (Figure 5B). Grip strength was measured after DEX administration. Compared with the control group, the DEX-treated group exhibited a significant 47% decrease in grip strength, whereas heat-killed postbiotic EF-2001 administration significantly increased grip strength by 13.5% at 3 mg/kg, 43.2% at 30 mg/kg, and 26.8% at curcumin group (Figure 5C). These results indicated that heat-killed postbiotic EF-2001 administration significantly improved muscle strength and counteracted muscle loss in the DEX-induced muscle atrophy model.

### 2.6. Effect of Heat-Killed Postbiotic EF-2001 on the Muscle Volume and Weight in Dexamethasone-Induced Muscle Atrophy in SD Rats

**Figure 6:**
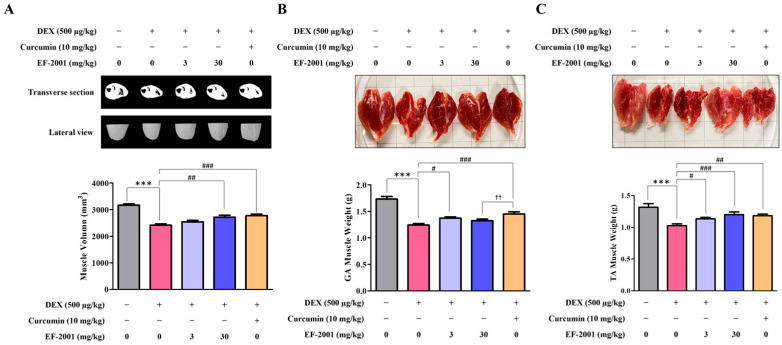
Effects of heat-killed postbiotic EF-2001 on the muscle volume and weight in DEX-induced muscle atrophy in SD rats based on micro-CT scans. (**A**) Muscle volume of the rats was measured using micro-CT after injection with DEX and absence or presence of heat-killed postbiotic EF-2001. Rats in each experimental group were administered heat-killed postbiotic EF-2001 for 2 weeks, then administered DEX for 5 d. Cinematic rendering is a 3D imaging technique for CT images. The image shows CT data obtained prior to sacrifice. Muscle volume determined using CT (BATMAN) score as a semiquantitative CTA-based grading system (**B**) Muscle tissue form and weight changes were determined for the gastrocnemius (GA) muscles after the sacrifice of experimental animal. (**C**) Muscle tissue form and weight changes were determined for the tibial anterior (TA) muscles after the sacrifice of experimental animal. Data are presented as means ± SEM (*n* = 15 per group). *** *p* < 0.001 compared with control group. ^#^ *p* < 0.05, ^##^ *p* < 0.01, ^###^ *p* < 0.001 compared with DEX-induced control group. ^††^ *p* < 0.01, EF 30 vs. Curcumin group. GA: gastrocnemius, TA: tibial anterior.

To establish a DEX-induced muscle atrophy model, male rats were divided into control (untreated) and DEX-treated groups. The rats were orally administered refined water or heat-killed postbiotic EF-2001 suspended in water once daily, according to the experimental schedule. DEX-treated groups were further divided into four subgroups (distilled water, 3 mg/kg EF-2001, 30 mg/kg EF-2001, and 10 mg/kg curcumin) to evaluate the effects of heat-killed postbiotic EF-2001 on muscle volume and weight. Muscle volume in the DEX group was lower than that in the control (CON) group, whereas treatment with 30 mg/kg heat-killed postbiotic EF-2001 and 10 mg/kg curcumin significantly prevented DEX-induced muscle loss (Figure 6A). To examine the correlation between the muscle-preserving effect and muscle mass, the weights of the gastrocnemius (GA) and tibialis anterior (TA) muscles were analyzed. These two muscles are critical in leg support, movement, and the maintenance of a stable gait. The GA muscle weight was significantly decreased by approximately 39.6% in the DEX group compared with the CON group, whereas the heat-killed postbiotic EF-2001 (3 mg/kg) group showed an increase of 10.4% and curcumin (10 mg/kg) group showed an increase of 16.7% (Figure 6B). Similarly, the tibial anterior (TA) muscle weight was significantly reduced by 28.2% in the DEX group; however, it significantly increased by 10.4% and 16.8% in the 3 and 30 mg/kg EF-2001-treated groups, respectively, and by 15.1% in the curcumin treated group (Figure 6C). These results indicated that heat-killed postbiotic EF-2001 exerted a clear protective effect against DEX-induced muscle loss in a dose-dependent manner.

### 2.7. Effect of Heat-Killed Postbiotic EF-2001 on Regulation of the Expression of Muscle Atrophy-Related Protein in Dexamethasone-Induced Muscle Atrophy in SD Rat Model

**Figure 7:**
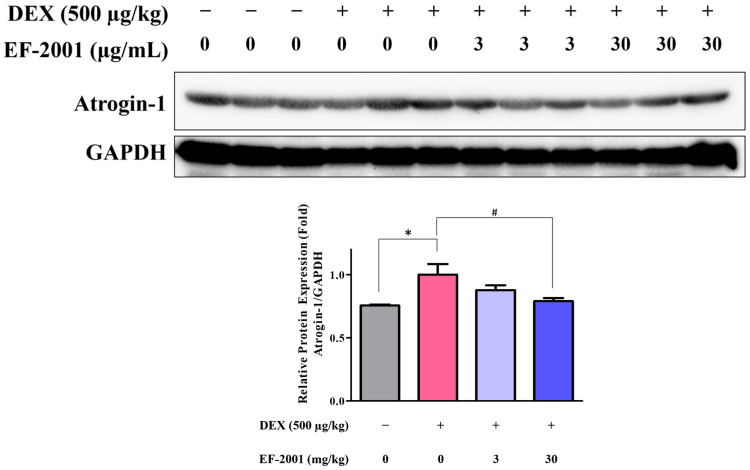
Effects of heat-killed postbiotic EF-2001 on expression of muscle atrophy related proteins with DEX-induced muscle atrophy in SD rats. Representative Western blot result of atrogin-1. Blots were quantified by densitometry and are expressed as relative to the control group. The protein expression of atrogin-1 on DEX-induced muscle atrophy in SD rats. SD rats were administrated with 3 or 30 mg/kg of EF-2001 for 2 weeks and tibial muscle atrophy induction was induced with DEX, in conjunction with heat-killed postbiotic EF-2001, for 5 d. The subjects were randomly selected within each group, and Western blotting was performed after lysis of the muscle tissue. Protein expression levels were normalized to GAPDH for quantification. Data are represented as mean ± SEM. * *p* < 0.05 vs. control group. ^#^ *p* < 0.05 vs. DEX-induced control group.

The effect of heat-killed postbiotic EF-2001 on the expression of muscle atrophy-related proteins in TA muscle tissues of normal and DEX-induced muscle atrophy in rat models was investigated. Western blot analysis was performed to assess the effect of heat-killed postbiotic EF-2001 treatment on the expression of muscle atrophy -related proteins, such as atrogin-1, under muscle atrophy conditions (Figure 7). Protein samples extracted from muscle tissues were analyzed by Western blotting. As evidenced by the results, atrogin-1 expression was significantly upregulated by 32.4% in the DEX-treated group, whereas it was significantly reduced in the heat-killed postbiotic EF-2001-treated groups, showing a decrease of 12.3% at 3 mg/kg and 21.1% at 30 mg/kg compared with the DEX group. These findings suggest that heat-killed postbiotic EF-2001 mitigates muscle atrophy by suppressing the expression of the muscle degradation-related gene, atrogin-1.

## 3. Discussion

DEX was administered to induce muscle atrophy models in C2C12 myotubes and SD rats [1,36,37,38,39,40]. Although the present study employed dexamethasone-treated C2C12 myotubes and rats, this model represents a pharmacological form of glucocorticoid-induced muscle atrophy rather than naturally occurring, age-related sarcopenia. Nevertheless, DEX-induced C2C12 myotubes are widely used to evaluate functional food materials aimed at improving muscle strength and mitigating muscle loss associated with sarcopenia [41,42,43]. Probiotics such as *Lactobacillus plantarum* TWK10 and *Bifidobacteria* enhance muscle strength [44,45,46]. Additionally, *Lactobacillus rhamnosus* JY02 and *Lactobacillus gasseri* IM13 protect against DEX-induced muscle damage [33,37,38]. The effects of heat-killed *Enterococcus faecalis* (EF-2001) on the length and area of Giemsa-stained differentiated C2C12 myotubes were examined (Figure 1).

Horse serum-supplemented medium induces myoblast differentiation in myotubes. This process plays a crucial role in the initial differentiation-induced growth of myotube length [34,35]. Our results demonstrated that heat-killed postbiotic EF-2001 increased the length and area of myotubes and protected them from DEX-induced damage (Figure 2).

Although the present study focused on the regulation of Atrogin-1 expression and AKT phosphorylation as key mechanisms underlying the anti-atrophic effects of EF-2001, the role of IGF-1 and mTOR signaling in EF-2001 mediated myotube growth warrants further investigation in future studies [47,48]. Comet analysis is a method for analyzing the degree of DNA damage based on the length of the tail moved by performing DNA staining for DEX-induced DNA fragmentation. Previous research on the inhibition of oxidative stress induced by radiation and DEX in astrocytoma cells involved examining DNA damage using a comet assay [49]. A protective effect against DEX-induced DNA damage in C2C12 myotubes was observed after pretreatment with heat-killed postbiotic EF-2001, as evidenced by the absence of damaged tails (Figure 1B).

AKT phosphorylation is hypothesized to be involved in the regulation of myotube growth via autophagy in C2C12 cells. Increased phosphorylation of AKT S473 reportedly activates autophagy signals in response to damage in muscle atrophy models [50,51]. In this study, the expression of phosphorylated AKT was increased at both residues by EF-2001 (Figure 3), supporting the hypothesis that AKT phosphorylation may contribute to muscle protein synthesis and cell survival in response to DEX-induced muscle atrophy. In addition, the expression of atrogin-1, a protein known to be upregulated in muscle atrophy, was downregulated following heat-killed postbiotic EF-2001 treatment. Materials containing *Lactobacillus paracasei* and *L. plantarum* HY7715 have also been reported to protect against muscle atrophy by downregulating atrogin-1 expression during C2C12 myotube differentiation [45,46,52]. In conclusion, heat-killed postbiotic EF-2001 suppressed the expression of atrogin-1, a related factor, in C2C12 muscle cells and DEX-induced muscle atrophy in rats (Figure 4 and Figure 7). An association between muscle atrophy and factors such as the myosin heavy chain, which utilizes the energy obtained from ATP hydrolysis for processes such as cytoplasmic division, vesicle transport, and cell motility in C2C12 muscle cells, has also been described [53]. These factors require further investigation.

Administration of heat-killed postbiotic EF-2001 protected against DEX-induced muscle atrophy in rats, as demonstrated in vitro. This protective effect was achieved through a reduction in the expression of atrogin-1. The degree of muscle atrophy in the experimental rats decreased after oral treatment with heat-killed postbiotic EF-2001 at doses of 3 and 30 mg/kg/day for 2 weeks, administered concurrently with DEX for 5 d. Similarly, curcumin, used as a reference compound, was orally administered at 10 mg/kg/day for 2 weeks, concurrently with DEX for 5 days under the same experimental conditions. Curcumin was selected as a reference compound based on previous evidence supporting its protective effects against muscle loss and its proposed utility in sarcopenia-related research [23]. In the DEX-induced muscle atrophy in vivo model, both heat-killed postbiotic EF-2001 and curcumin treatments led to a significant improvement in grip strength, with heat-killed postbiotic EF-2001 showing a dose-dependent effect (Figure 5C). Quantitative muscle loss and volumetric muscle reduction were confirmed by transverse section and lateral view analyses using micro-CT across all groups (Figure 6A). Novel results were obtained by isolating and weighing the two muscle types (GA and TA) in the tibial region of each group (Figure 6B,C). Other studies have reported prevention of the loss of larger muscles, such as the GA and TA, in the tibial region. This study used a muscle atrophy model in SD rats, which have relatively large muscles in the tibial region [18,19]. Statistically significant changes were observed in the GA and TA muscles in groups treated with heat-killed postbiotic EF-2001 or curcumin, which counteracted DEX-induced muscle loss. Furthermore, as mentioned above, research should focus on overall muscle mass and functional grip strength in the tibial region, where muscle loss was effectively prevented in the heat-killed postbiotic EF-2001 treated group, as shown in Figure 5 and Figure 6. Although the 3 mg/kg EF-2001 group exhibited statistical significance in grip strength measurements, the magnitude of functional recovery was modest compared with the 30 mg/kg group. This discrepancy highlights the distinction between statistical significance and physiological relevance, suggesting that higher doses may be required to achieve meaningful functional improvement under DEX-induced muscle atrophy conditions.

These results suggest that orally administered heat-killed postbiotic EF-2001 effectively protects against DEX-induced muscle atrophy and loss. In this study, treatment with heat-killed postbiotic EF-2001 resulted in AKT phosphorylation, leading to the activation of the muscle protein synthesis pathway in C2C12 myotube. Furthermore, the regulation of the expression of atrogin-1 through the intake of heat-killed postbiotic EF-2001 in vivo were consistent with the in vitro study results, providing further support for this hypothesis (Figure 7). However, further research is required to examine the changes in intracellular signaling induced by EF-2001 treatment during muscle cell differentiation.

EF-2001 is derived from infant feces and supplied in a heat-treated, desiccated powder form, where microorganisms are inactivated [13,54]. Heat-killed postbiotic EF-2001 exerts beneficial effects on human health, including antioxidant, anti-allergic, anti-inflammatory, antitumor, and immunomodulatory effects [18,20,21,26,55,56,57]. However, intestinal infection with *Enterococcus faecalis* could pose a threat, as it is associated with aggravating symptoms of enteritis and Crohn’s disease [8,10,28,30]. Nevertheless, the intracellular antioxidant effect of heat-killed *Enterococcus faecalis* has been reported in many studies [12,31].

The dose of heat-killed postbiotic EF-2001 used in this experiment was based on that administered to humans. The adult clinical dose of heat-killed postbiotic EF-2001 was 1.5 g/session once daily. When converted to an adult weight of 60 kg, the clinical dose was 25 mg/kg/d. For rats, the recommended daily dose was 307.5 mg/kg/d, 12.3 times that for human adults. The toxicity test results of oral administration indicated that the estimated 50% lethal dose (LD50) of heat-killed postbiotic EF-2001 for male and female rats is likely to be greater than 5000 mg/kg body weight/day [14,17,50]. In this study, toxicity tests of heat-killed postbiotic EF-2001 were not performed; however, the maximum dose administered was 30 mg/kg. This concentration is much lower than that used by other research groups and is within the safe range based on toxicity results from other studies. Reducing atrogin-1 expression by heat-killed postbiotic EF-2001 may provide protection against pharmacologically induced muscle atrophy. However, further studies are required to determine the relevance of these findings to age-related sarcopenia and other muscle-wasting conditions. This study was limited to a single postbiotic strain, heat-killed *Enterococcus faecalis* EF-2001, and did not include comparisons with other strains or an evaluation of its interaction with the gut microbiota. In addition, the relatively short duration of the intervention warrants further investigation into the long-term safety and toxicity of EF-2001. Future studies should address these limitations through multi-strain comparative analyses, exploration of gut–muscle axis–related mechanisms, and well-designed clinical studies to support potential human applications.

## 4. Materials and Methods

### 4.1. Preparation of Heat-Killed Postbiotic E. faecalis EF-2001

Heat-killed *E. faecalis*, known as EF-2001, is a merchantable-quality postbiotic purified from human feces, which is supplied as a heat-killed, dried powder by bereum Co., Ltd. (Wonju, Republic of Korea). Dried EF-2001 contains 7.5 × 10^12^ cells per gram prior to being heat-killed.

### 4.2. Preparation of the Reference Compound Curcumin

Curcumin was used as a positive control due to its well-established anti-atrophic effects and its recognized relevance in sarcopenia and muscle atrophy research [24]. Curcumin (≥94% purity; Sigma-Aldrich, St. Louis, MO, USA) was used as the reference compound in this study [15,58]. The reference solution was freshly prepared by suspending curcumin powder in distilled water (DW) before administration. Curcumin was orally administered to the experimental animals at a dose of 10 mg/kg/day.

### 4.3. Cell Culture and Differentiation

Mouse skeletal muscle C2C12 cells were purchased from American Type Culture Collection (Manassas, VA, USA). C2C12 myoblasts were cultivated in Dulbecco’s modified Eagle’s medium (DMEM) supplemented with 10% (*v*/*v*) fetal bovine serum and 1% (*v*/*v*) streptomycin and penicillin, at 37 °C in a humidified atmosphere with 5% CO_2_. C2C12 myoblasts were seeded in a 6-well plate (1 × 10^5^ cells/mL). To induce differentiation into myotubes, the medium was replaced with differentiation medium when C2C12 myoblasts became 80% confluent; the differentiation medium was supplemented with 2% (*v*/*v*) horse serum and 1% (*v*/*v*) streptomycin and penicillin. The cells were then incubated with heat-killed postbiotic EF-2001 (50, 100, 250, and 500 μg/mL) for 48 h. The in vitro concentrations of EF-2001 (50–500 μg/mL) were selected based on previous cytotoxicity studies demonstrating no adverse effects within this range.

### 4.4. Induction of Muscle Atrophy in C2C12 Myotubes by Dexamethasone

After seeding C2C12 cells in 6-well plates at a density of 1 × 10^5^ cells/mL, the cells were differentiated into myotubes using differentiation medium when they reached approximately 100% confluence. The differentiation medium was refreshed on days 0, 2, and 4. Heat-killed postbiotic EF-2001 was added to the differentiation medium at concentrations of 50, 100, 250, and 500 μg/mL. After myotube differentiation, muscle atrophy was induced by treatment with 100 μM dexamethasone (DEX; Sigma-Aldrich, St. Louis, MO, USA) for 24 h. The DEX-induced myotube atrophy protocol was performed on day 7.

### 4.5. Giemsa Staining

Differentiated C2C12 cells were stained with Giemsa staining solution 5% (*v*/*v*). The Giemsa staining working solution was prepared by diluting the Giemsa staining stock solution (GSS500; Scytek, Logan, UT, USA) in a 1:20 ratio in phosphate-buffered saline (PBS; pH 5.6). The cells were washed twice with PBS. To fix the cells, 100% methanol was added and the solution was allowed to stand at room temperature (25 ± 3 °C) for 5 min. Methanol was removed by aspiration and the cells were dried for 10 min. The Giemsa stain working solution was added (1 mL/well) for 20 min and then removed by aspiration. The cells were washed thrice with PBS, dried, and stored at room temperature. Cells were photographed using a Nikon inverted microscope (Nikon, Tokyo, Japan) at 100× magnification, and myotube length, width, and area were measured using ImageJ 64bit Java v1.8.0.170 software (National Institutes of Health, Frederick, MD, USA).

### 4.6. Comet Assay

The cell suspension was mixed with 0.3% low melting agarose (LMA) at 37 °C, and the mixture was laid out on fully frosted microscope slides (precoated with 1% normal melting agarose) and solidified for 5 min at −20 °C). After solidification of the agarose, the slide was covered with 0.5% LMA and then immersed in a lysis solution (2.5 M NaCl, 100 mM Na-EDTA, 10 mM Tris, 1% Triton X-100, and 10% DMSO; pH 10) for 1 h at 4 °C. The slides were then placed in a gel electrophoresis apparatus containing 300 mM NaOH and 10 mM Na-EDTA (pH 13) for 40 min to allow for DNA unwinding and expression of alkali-labile damage. Subsequently, an electrical field was applied (300 mA, 25 V) for 20 min at 4 °C to draw the negatively charged DNA toward the anode. After electrophoresis, slides were washed thrice for 5 min at 4 °C in a neutralizing buffer (0.4 M Tris, pH 7.5), followed by staining with 20 μg/mL propidium iodide (PI; Sigma-Aldrich). Slides were then examined under a fluorescence microscope (Carl Zeiss, Oberkochen, Germany). The comet assay was performed as previously described [16,19,22,51,52,53].

### 4.7. Western Blotting

To analyze protein expression in vitro and in vivo, C2C12 myoblasts and muscle tissues from DEX-treated rats were prepared for Western blot analysis. At the indicated time points (days 2–7), cells and muscle tissues were lysed using lysis buffer (iNtRON Biotechnology Inc., Sungnam, Republic of Korea), and protein concentrations were quantified for Western blotting. After sonication, proteins were quantified using the Bradford assay (Bio-Rad, Hercules, CA, USA) for Western blotting. The SDS-PAGE ratio was determined based on the molecular weight (kDa) of the protein to be confirmed, and electrophoresis was performed at 100 V for approximately 2 h. The following antibodies were used: anti-p-AKT (S473) (1:1000; Cell Signaling Technology, New England Biolabs, Ipswich, MA, USA; #9271), anti-AKT (1:1000; Cell Signaling Technology; #9272), anti-atrogin-1 (anti-FBX32) (1:1000; Abcam, Cambridge, England; sc-166806), and anti-GAPDH (1:1000; Cell Signaling Technology; #5174). The membrane was washed three times with Tris-buffered saline containing Tween 20 for 10 min, and secondary antibodies were added at a ratio of 1:5000 for 2 h at room temperature. The transferred protein band on the PVDF membrane was measured using an LAS 4000 system (GE Healthcare, Little Chalfont, UK) by inducing an enhanced chemiluminescence reaction.

### 4.8. Animal Experiments and Treatments

Male Sprague Dawley rats (6-week-old) were purchased from Samtako Inc. (Osan, Republic of Korea) and maintained at constant temperature (21 ± 1 °C) and humidity (45 ± 10%) with a 12 h light/dark cycle. The rats were provided with a solid feed (Rodent feed; Purina Co., Ltd., Yangpyeong, Republic of Korea). After a one-week adaptation period, the rats were divided into five groups (*n* = 15 each): control group (CON), DEX-treated group (DEX), low-dose heat-killed postbiotic EF-2001 (3 mg/kg) group (EF 3), high-dose heat-killed postbiotic EF-2001 (30 mg/kg) group (EF 30), and curcumin (10 mg/kg) group (CUR).

Muscle atrophy was induced by subcutaneous injection of DEX (500 μg/kg) once daily for 5 d. EF-2001 or curcumin was administered prior to the initiation of dexamethasone treatment and was co-administered throughout the DEX treatment period. EF-2001 and curcumin were diluted in ddH_2_O and administered orally at 12 a.m. at 400 μL for 2 weeks and 5 days. CON- and DEX-treated rats were orally administered the same volume of ddH_2_O. All animal procedures and protocols were conducted in accordance with the Guidelines for Animal Experimentation of the National Institutes of Health Guide for the Care and Use of Laboratory Animals, and were approved by the Yonsei University Mirae Campus Institutional Animal Care and Use Committee (approval number: YWCI-202309-021-01).

### 4.9. Grip Strength Test

The grip strength of the rats were determined using a grip strength meter (Jeongdo Bio & Plant, Co., Seoul, Republic of Korea). Briefly, to test grip strength, the tails of the rats were held such that their forelimbs could grasp the wire mesh. The rats were then gently pulled backward until their grips were released. The grip strength test was performed three times for each rat on the following days: before sample administration, 2 weeks after sample administration, after induction of muscle atrophy, and immediately before sacrifice.

### 4.10. Measurement of Muscle Volume Using Micro-Computed Tomography

Micro-computed tomography (micro-CT) image data were obtained from day 0 to day 5 after DEX-induced muscle atrophy and before sacrifice (on day 6 after dexamethasone treatment by intraperitoneal [I.P.] injection) using a SkyScan 1076 micro-CT (Bruker, Bremen, Germany) at a resolution of 30 μm, with the following parameters: 100 kV, 100 mA, 790 ms, and a rotation step of 1.2°. All rats were anesthetized during rotation and scanning. Beam-hardening errors were corrected to improve image quality using flat-field correction before scanning and during reconstruction. To evaluate muscle volume, two-dimensional and three-dimensional models of the muscles were generated using a CT-Analyzer 1.11 (Bruker). Muscle volume was measured using the N-recon CTAn software (version 2.0, Bruker).

### 4.11. Statistical Analysis

Statistical analyses were performed using SAS statistical software (version 9.4, SAS Institute, Cary, NC, USA). Multiple group data were analyzed using one-way analysis of variance followed by Dunnett’s multiple range test. Comparisons between two groups were performed using *t*-tests. All results are expressed as mean ± standard error of mean.

## 5. Conclusions

EF-2001 has emerged as a promising probiotic and postbiotic with genomic traits that enhance gut health without producing harmful biogenic amines. Its favorable safety profile, marked by minimal virulence, low d-lactate production, and broad antibiotic susceptibility, makes it a strong candidate for therapeutic use, although vigilance is required regarding its resistance to specific aminoglycosides. The resilience of the strain in simulated gastrointestinal environments and the absence of bile salt deconjugation and hemolytic activity further underscore its probiotic and post-biotic potential. Beyond gut health, EF-2001 has the potential to address broader health concerns, including metabolic conditions such as obesity and diabetes, as well as mental health disorders influenced by the gut microbiota.

Future investigations should focus on heat-killed postbiotic EF-2001’s interactions between the human microbiome, their long-term health impacts, and their potential for chronic disease prevention. Furthermore, their application in functional foods, dietary supplements, and pharmaceuticals could pave the way for personalized and effective health solutions. As the field of microbiome research evolves, EF-2001 is expected to contribute to the next generation of probiotic and postbiotic therapies.

**Figure 8:**
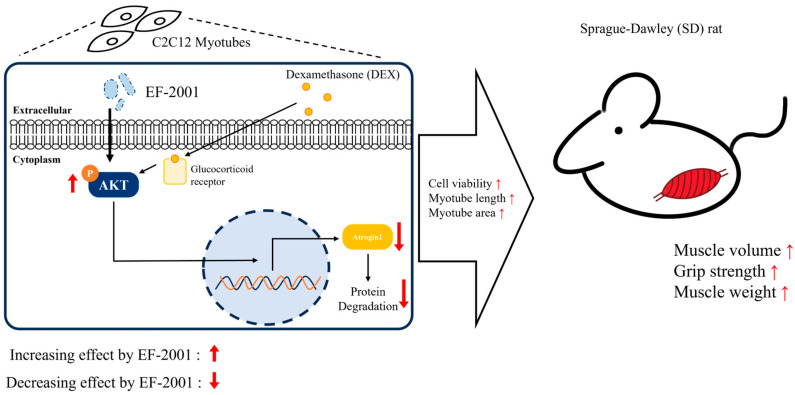
Schematic representation illustrating the proposed mechanisms by which heat-killed postbiotic EF-2001 modulates muscle atrophy in DEX-induced C2C12 myotubes and sarcopenic muscle atrophy in SD rat model.

Additionally, this study highlights EF-2001’s muscle-preserving effects, particularly in a DEX-induced muscle atrophy model. Heat-killed postbiotic EF-2001 mitigated the reduction in muscle weight, grip strength, volume, and mass in DEX-treated SD rats while increasing the phosphorylation of AKT at residue S473, thereby suppressing muscle damage signals (Figure 8). Notably, curcumin, which was used as a reference compound, also alleviated DEX-induced muscle atrophy. However, heat-killed postbiotic EF-2001 exhibited comparable efficacy, suggesting that its postbiotic-derived bioactivity may provide an alternative therapeutic strategy for muscle preservation. These findings suggest that heat-killed postbiotic EF-2001 regulates muscle atrophy by targeting protective signaling pathways during muscle cell differentiation, thus offering a promising therapeutic approach for muscle preservation.

The current study established foundational safety data for heat-killed postbiotic EF-2001 and demonstrated its protective effects against dexamethasone-induced muscle atrophy in animal experiments. These findings support further investigation of EF-2001 in human studies to explore its potential applications as a functional food ingredient related to metabolic and gut health. Planned clinical studies are intended to support an application for functional health food approval by the Ministry of Food and Drug Safety (MFDS). Future research will focus on addressing the limitations of the present study and further characterizing the biological functions of heat-killed postbiotic EF-2001.

## Data Availability

The original contributions presented in this study are included in the article. Further inquiries can be directed to the corresponding author.

## Abbreviations

The following abbreviations are used in this manuscript:

AKT	Protein Kinase B
CON	Control group
DEX	Dexamethasone
DMEM	Dulbecco’s Modified Eagle’s Medium
EF-2001	Heat-killed *Enterococcus faecalis* EF-2001
GAPDH	Glyceraldehyde 3-phosphate dehydrogenase
GA	Gastrocnemius
GS	Grip strength
GSM	Grip strength meter
Micro-CT	Micro-Computed Tomography
MTT	3-(4,5-Dimethylthiazol-2-yl)-2,5-diphenyltetrazolium bromide
p-AKT	Phospho-AKT
PI	Propidium Iodide
OD	Optical density
PBS	Phosphate-buffered saline
RT	Room temperature
SD	Sprague–Dawley
SDS PAGE	Sodium Dodecyl Sulfate Polyacrylamide Gel Electrophoresis
TA	Tibialis anterior
